# Phylogenetic Utility of rRNA ITS2 Sequence-Structure under Functional Constraint

**DOI:** 10.3390/ijms21176395

**Published:** 2020-09-03

**Authors:** Wei Zhang, Wen Tian, Zhipeng Gao, Guoli Wang, Hong Zhao

**Affiliations:** 1 Marine College, Shandong University, Weihai 264209, China; gaozhipeng@mail.sdu.edu.cn (Z.G.); wangguoli1996@sina.com (G.W.); zhaohong@sdu.edu.cn (H.Z.); 2 State Key Laboratory of Ballast Water Research, Comprehensive Technical Service Center of Jiangyin Customs, Jiangyin 214440, China; tmdingding@163.com

**Keywords:** compensatory base change, phylogeny, ribosome biogenesis, ribosomal ITS2, secondary structure

## Abstract

The crucial function of the internal transcribed spacer 2 (ITS2) region in ribosome biogenesis depends on its secondary and tertiary structures. Despite rapidly evolving, ITS2 is under evolutionary constraints to maintain the specific secondary structures that provide functionality. A link between function, structure and evolution could contribute an understanding to each other and recently has created a growing point of sequence-structure phylogeny of ITS2. Here we briefly review the current knowledge of ITS2 processing in ribosome biogenesis, focusing on the conservative characteristics of ITS2 secondary structure, including structure form, structural motifs, cleavage sites, and base-pair interactions. We then review the phylogenetic implications and applications of this structure information, including structure-guiding sequence alignment, base-pair mutation model, and species distinguishing. We give the rationale for why incorporating structure information into tree construction could improve reliability and accuracy, and some perspectives of bioinformatics coding that allow for a meaningful evolutionary character to be extracted. In sum, this review of the integration of function, structure and evolution of ITS2 will expand the traditional sequence-based ITS2 phylogeny and thus contributes to the tree of life. The generality of ITS2 characteristics may also inspire phylogenetic use of other similar structural regions.

## 1. ITS2 Processing in Ribosome Biogenesis

**Figure 1:**
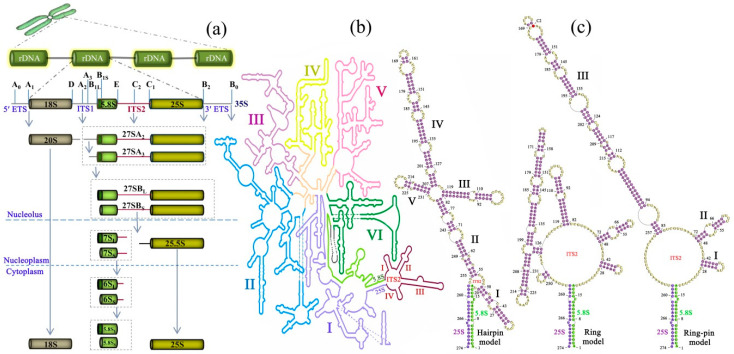
Overview of the ITS2 (internal transcribed spacer 2) processing and folding in the ribosome biogenesis of the yeast *S. cerevisiae*. (**a**) ITS2 location and processing in pre-rRNA from genome to transcriptome. ITS2 and ITS1 are intercalated in the 18S–5.8S–25S tandem arrays, separating the elements of the pre-rRNA. The endonucleolytic cleavage sites are labelled A–E on the pre-rRNA. ITS2 region is highlighted in dark red in each processing state. (**b**) ITS2 location in the secondary structure of the 5.8S/25S pre-RNA. The ITS2 and 5.8S are displayed in dark red and bright green, respectively; the six typical domains of 25S are labelled I–VI in distinct colors. (**c**) The three proposed secondary structure models for ITS2. The C_2_ cleavage site is highlighted in red in stem III of the ring-pin model. The secondary structure scheme is taken and modified from *S. cerevisiae* LSU (http://apollo.chemistry.gatech.edu/RibosomeGallery/).

Ribosome, the ribonucleoprotein nano-machinery that translates genetic codes into amino acids, is indispensable to all life forms. The eukaryotic ribosome of the model organism *Saccharomyces cerevisiae* is made up of 40S small subunit (SSU) and 60S large subunit (LSU). SSU is formed by 18S rRNA and 33 ribosomal proteins (RPs), and LSU is formed by 25S, 5.8S, 5S rRNA as well as 46 RPs [1]. Ribosome biogenesis is a highly complex process involving folding, modifying, processing, assembling and transporting of the precursor rRNAs (pre-rRNAs), orchestrated by more than 200 assembly factors (AFs). Such a complicated process begins in the nucleolus with the massive transcription of ribosomal DNA (rDNA) by RNA polymerase I, accounting for approximately 60% of total cellular RNA. rDNA exists as hundreds to thousands of tandemly repeated copies at one or more chromosomal locations [2]. These copies become homogenized after concerted evolution via unequal crossing over or gene conversion. Each copy is a fundamental transcript unit and is first transcribed into a 35S pre-rRNA, consisting of pre-rRNA of the 18S, 25S and 5.8S rRNA. These regions are flanked by 5′ and 3′ external spacers (ETS) and are separated by two internal transcribed spacers, ITS1 and ITS2 (Figure 1a).

Removal of ITS2 is the longest-lasting step during LSU synthesis and is considered essential to make functional ribosome [3]. ITS removal begins with the cleavage of the ITS1 A2 (or A3) site and generates the 20S and 27SA2 (or 27SA3), separating the two parallel synthesis pathways of SSU and LSU. In the LSU pathway, the subsequent trimming of the 5′ end of 27SA2/A3 produces two types of intermediates, 27SB_L_ and 27SB_S_, based on their different exonucleolytic cleavage sites (Figure 1a). The 27SB_S/L_ is assembled in nucleolus and then transited to nucleoplasm for subsequent processing. The ITS2 cleavage starts at the C2 site in 27SB_L/S_ by endonuclease Las1, and generates 25.5S and 7S_L/S_ pre-rRNAs. Then, the 5′ end of 25.5S pre-rRNA is phosphorylated by polynucleotide kinase Grc3 and subsequently trimmed by nucleases Rat1 and Rai1to create 25S [4,5]. In contrast, the 7S_L/S_ pre-rRNAs are trimmed twice from 6S_S/L_ to 5.8S_L/S_, which occurs sequentially by exosome in nucleoplasm and then by NgI2–Rex1-3 complex in cytoplasm [6]. Notably, all of these ITS2 processing steps are tightly coupled with diverse nucleases, ribosomal proteins and assembly factors, which assemble in a hierarchical and coordinated way [7,8]. Specifically, at the 27SB particle state, three ITS2-binding AFs (Nop15, Cic1 and Rlp7) and nucleolar protein Nop7, Nop53 together form a characteristic ‘foot’ structure, which was recently shown by cryo-electron microscopy (cryo-EM) [8,9,10,11].

## 2. ITS2 Function in Ribosome Biogenesis

The intriguing question is why the processing of ITS2 cleavage is so complicated rather than parsimoniously excising both endpoints of 27SB pre-rRNA all at once? The final products of ITS2 cleavage, 25S and 5.8S, may provide some clues to this question. The 5′ end of 25S rRNA and the 3′ end of 5.8S rRNA do not join in the mature 60S subunits but interact together to form a characteristic base-paired proximal stem, which is evolutionarily conserved among eukaryotes (Figure 1b). Both the cryo-EM and the recent high-throughput RNA structure probing method indicate that the 5.8S-25S base-paired proximal stem occurs as early as the co-transcription of pre-rRNAs [7]. Site-specific mutagenesis that changes this 5.8S–25S base-paired stem in either strand of 5.8S or 25S can inhibit the downstream processing [12]. In contrast, an alternative compensatory double mutation can remove this inhibition to some extent [12]. Clearly, these functional analyses demonstrate that this ITS2 proximal stem is critical in ribosome biogenesis. Given that ITS2 is the last and longest-lasting spacer removed during ribosome biogenesis, the 5.8S-ITS2-25S complex (27SB pre-rRNA) is the longest-lived pre-rRNA processing intermediate [13]. To maintain stability, 27SB pre-rRNA, by itself, can rapidly fold into secondary or tertiary structural domains, in which the ITS2 folding could bring 5.8S and 25S pre-rRNA into close vicinity, thus facilitating their hybridization (Figure 1b). Furthermore, the tertiary architecture of the 27SB rRNA complex is dynamic, wherein conformational rearrangement is essential for subsequent biogenesis assembly [7,14]. In this situation, ITS2 and its associated AFs are thought to function together as a scaffold to mediate topological rearrangements or as a timer to prevent premature folding [13]. Consistent with this idea, studies based on cryo-EM showed that 5.8S, ITS2, and 25S domains I and II, fold and assemble initially into a rigid core particle forming an exoskeleton for further assembly [9,11]. Given that any sequence change could trigger conformational switches, it is reasonable to presume that ITS2 removal in such a hierarchical way is coupled with the dismantling of a hallmark transient foot structure, to drive or maintain the intermediate conformation to a correct architecture [3].

## 3. Characteristics of ITS2 Secondary Structure

The first acknowledged ITS2 secondary structure was determined in *S. cerevisiae* based on chemical and enzymatic probing together with minimum free-energy calculation of the in vitro synthesized 37S pre-rRNA [15]. In this structure, ITS2 sequence folds extensively through long-range interaction adjacent to the 5′ end of 25S and the 3′ end of 5.8S, yielding five tightly base-paired helixes, which was later known as the “hairpin model” (Figure 1c). Subsequent verification tests in vivo showed that site-directed mutations or deletions on different sites of this structure could completely or partially block the maturation of 25S rRNA [12,16,17]. These findings supported the ITS2 “hairpin model” and showed its distinct functional components.

Several experimental methods in vivo, such as cryo-EM, site-directed mutagenesis and chemical and structural probing are reliable to infer RNA secondary structures, but they are laborious and are not suitable for large dataset analyses. Alternatively, the most conventional method is use of thermodynamic energy optimization to predict secondary structures and then use phylogenetic comparative analysis to examine common structures from closely related lineages. Specifically, as more and more ITS2 structural features become available in databases [18], ITS2 secondary-structure predictions will be based not only on the nucleotide sequence but also on the templates for homology modeling, in which subtle structural motifs that rarely automatically fold by standard minimum free energy (MFE) approach are preset [19].

Unexpectedly, in silico prediction from a broad range of eukaryotic diversity has yielded two more ITS2 secondary-structure models, which are distinct from the “hairpin model” being found in yeast and a few other species. One is the “ring model”, which is detected mainly in vertebrates [12,20]. In this model structure, the 5′ to 3′ long-range interactions in the “hairpin model” disappear, wherein each strand folds by itself into two separate stems instead. In addition, the longest stem always shares a common short base-stem with another stems, thereby characterized as “giant stem with lateral branches” (Figure 1c). Collectively, four or five extended stems in this structure radiate from an open central core or ring.

The third ITS2 secondary-structure model termed “four-helix model” [21] or “ring-pin model” [7], is widely observed throughout the eukaryote [22,23,24]. In this structure, the longest stem of the ring model and its two neighboring stems merge together into a much longer stem. Therefore, the “ring-pin model” is typically recognizable as a much longer helix together with three relatively short helices radiating from an open central ring. Notably, not all the eukaryotes have the fixed four helices because the helix I and helix IV are most variable and may disappear in some organisms (Figure 1c). In contrast, the longest helix III and its neighboring helix II are more stable and common to all eukaryotes. The basal pairing of the helices I and helix II are usually base-paired conserved and serve as a scaffold for shaping the structure. The most conserved component of helix III at the tip of the helix, and in land plants a hallmark motif UGGU, exist in this region [21,22].

## 4. Dynamic Model of ITS2 Secondary Structure

Cote et al. first examined the occurrence and functional significance of the distinct ITS2 structures between the “hairpin model” and “ring model” using functional genetic assay [25]. Unexpectedly, they found both model structures are necessary in vivo. Given their different effects on downstream processing, Cote et al. hypothesized that the “ring model” structure promotes AFs binding and is required in earlier pre-rRNA complex assembly, and is then followed by an induced “zipping up” transition to the “hairpin model” structure that facilitates formation of the ITS2 proximal stem. This idea is supported by the recent high-resolution cryo-EM structures of pre-60S particles which give convincing evidence that ITS2 function through conformational changes [9,10]. This process is also coordinated with stepwise binding or separating of AFs, by which the transient structure complex can be stabilized or flexible to another change [3]. In contrast, Burlacu et al. monitored rRNA restructuring processing events from 35S to 27SB using a high-throughput RNA structure probing method (ChemModSeq15), by which they quantified the nucleotide flexibilities of ITS2 structure [7]. The result showed that ITS2 generally maintains a conserved structure, which agrees best with the “ring-pin model”. Thus, it remains largely elusive whether ITS2 functions with a fixed or dynamic conformational model.

Although each model has been confirmed by alternative methods, the current three ITS2 secondary-structure models are more or less derived from minimum free energy folding (MFE). Actually, ITS2 does not fold and function by itself but is mutually interdependent with dozens of AFs that function as chaperones and scaffolds to direct and stabilize ITS2 folding [7,8,11]. It is reasonable to speculate that the final ITS2 folding shape does not necessarily follow the law of thermodynamics. It is not surprising that the proposed structure in vivo is rarely automatically yielded by standard MFE folding. Despite recent advances in cryo-EM that have provided detailed portions of ITS2 secondary-structure information [10], it is still challenging to get a comprehensive ITS2 secondary structure from the snapshots of a number of assembly intermediates.

## 5. ITS2 Conserved Structure and Sequence-Structure Alignment

Sequence alignment is essential for almost every sequence-based molecular science. The traditional alignment methods are mainly based on the similarity of DNA sequences which are weighted by the score of substitution versus indel [26]. However, sequence alone is not enough for RNA. Under functional constraint, RNA structure is always more conserved than sequence. Homologous RNA sequences that function equally as binding, cleaving or catalyzing may have distinct nucleotides, challenging the traditional algorithm of sequence-based alignment. If the common structures of these homologous sequences are taken into account in RNA folding and alignment, the reliability of the results can be greatly improved [27,28]. On the other hand, calculating a common structure of a given set of RNA molecules can predict the core elements in function [29,30].

In particular, ITS2 conserved structure has been intensively explored and used. An increasing number of ITS2 sequences and their known secondary structures have been deposited in the special ITS2 database [18] as templates for ITS2 constraint folding and sequence-structure alignment [31]. Comparative analysis of more than 54,000 currently known ITS2 sequence-structures in the ITS2 database by the homology-modeling method indicates a common core of the “ring-pin” model structure throughout the eukaryote [22]. The 4SALE program is specially developed to use this “ring-pin” model as the constraint for a fast and synchronous ITS2 sequences and secondary structures alignment [32]. These “ring-pin” structures are not thermodynamically optimal in most cases, owing to the reduction of some possible base pairs by the homology-modeling method. However, these common core structural elements in the “ring-pin” structure act as anchors during the 4SALE alignment process [33]. In addition, the 4SALE program aligns an ITS2 sequence-structure matrix based on a 12-letter string that codes for four nucleotides with three states in a secondary structure (unpaired, paired left or right), wherein the ITS2 specific scoring matrix for substitutions and gap costs are implemented in alignment calculating [32,33]. Based on the common core “ring-pin” structural elements and the ITS2 specific scoring matrix, it is much easier to align the similar structural elements among distinct evolutionary lineages [33].

## 6. Structure-Guiding Alignment for Phylogeny

Sequence alignment is a representation of evolutionary history rather than the work of mathematics and computing. Molecular structures could also contain evolutionary information based on the speculation that structurally related genes are more likely to have derived from a common ancestor than have arisen independently [34]. Thus, sequence similarity, together with their common structures and shared functional constraints, are all considered as homology [35]. Actually, taking the biological criterion of sequence-structure-function relationship into alignment procedure is considered more reliable than using mathematical criteria alone [36], especially when multiple equally optimal alignments occur in mathematical criteria.

rDNAs have intrinsic appeal for phylogenetic inference because they occur in all prokaryotic and eukaryotic organisms and thus have the potential to compare characters common to all of life. For example, Woese and Fox’s pioneering work with 16S rDNA was a milestone in understanding the fundamental tree of life [37]. The ribosomal ITS2 sequence evolves very quickly, making it an excellent marker that is widely used in low level phylogenetic analyses and DNA barcoding [38,39]. However, this hyper-variable feature also poses a barrier for application to deeper phylogeny. The secondary structure of ITS2 could provide a solution to this problem because it is more conserved than sequence and can be used for high divergent-sequence alignment based on its structural constraints or homologous locations [40]. For example, the species composition of the Chinese herb Mutong includes four families with only 56–70% nucleotide identity, indicating that it is difficult to align accurately using sequence similarity alone. Fortunately, they are successfully aligned by ITS2 structure guiding [41]. Furthermore, ambiguous or unalignable regions are always cut from traditional phylogenetic analyses. If these regions could be successfully aligned using structure guidelines, they would provide additional phylogenetic information and thus improve accuracy and robustness of phylogenetic trees. Therefore, the combination of the ITS2 fast evolving sequence with its highly conserved structure makes ITS2 as an excellent “double-edged” tool for eukaryote evolutionary comparisons [21].

## 7. CBC in ITS2 Secondary Structure

As mentioned above, ITS2′s function relies on its secondary or tertiary structures which are formed by base-pair interactions. Mutations occurring on any base within this interaction may disturb their structure and thus hinder their normal function. Therefore, it is intriguing to make clear how the fast evolving ITS2 can maintain its structure to perform normal functions. Compensatory base change (CBC) or compensatory mutation, wherein a base substitution on one side (hemi-CBC) is compensated by a substitution on the other side of that base pair, is expected to restore base-pairing and maintain the structure [42]. From an evolutionary perspective, such CBCs can be reserved in an ancestral ITS2 structure and are inherited by its descendant, making structural states include unique phylogenetic information not found in the nucleotide sequence [43].

The ITS2 stems are frequently maintained by base-pair interactions between the four canonical Watson-Crick (WC) base pairs, but the remaining 12 noncanonical base pairs also occur occasionally [44]. Of these noncanonical base pairs, purine–purine and pyrimidine–pyrimidine combinations are rarely formed due to their unstable binds, and selective constraints generally limit such mismatch occurrences. However, the purine–pyrimidine base-pair GU, whose stability is less than WC pairs but higher than all other noncanonical base pairs, is also thought to frequently occur [45]. GU is thus taken account into some analyses such as structure prediction and molecular evolution [46,47] together with WC. Recently, the other purine–pyrimidine AC was also observed in ITS2 secondary structure. Compared with GU, AC has lower frequency but higher mutability and generally has a similar role as GU in compensatory mutations [42].

**Figure 2:**
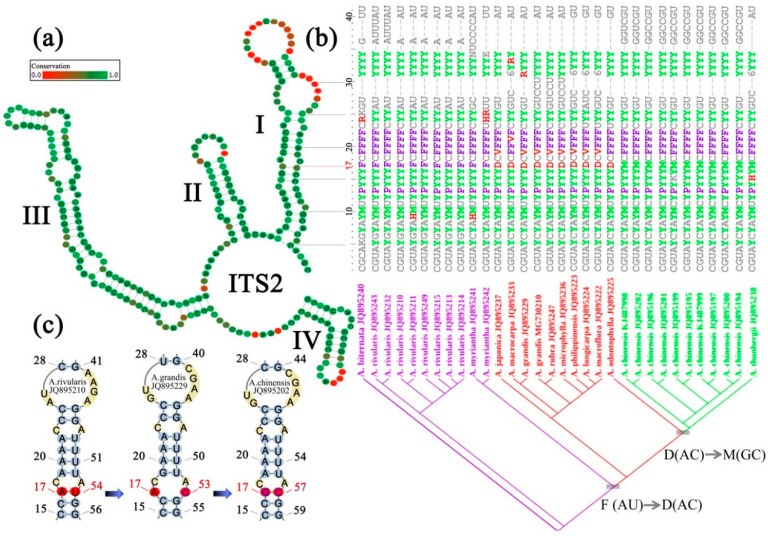
Transformation, visualization and optimization of compensatory base change in ITS2 secondary structure. (**a**) An ITS2 consensus secondary structure derived from closely related species of *Astilbe*. The four stems are labelled I–IV. Degree of site conservation over the entire alignment is displayed in colored grades from green (conservative) to red (variable). (**b**) A transformed coding matrix of sequence-structure information from portion of ITS2 stem I region. Base-pair information of ITS2 secondary structure (**a**) is coded and merged into a single character by using a modified 28-symbol coding matrix [40], wherein characters involved in CBC (compensatory base change) and hemi-CBC substitutions are highlighted in different colors, i.e., UA(P)-UG(R)-CG(Y), UA(P)-CA(H)-CG(Y), AU(F)-GU(V)-GC(M), AU(F)-AC(D)-GC(M). Character positions in the transformed matrix are corresponding to the ITS2 stem I one by one. Species of each sequence and their phylogenetic relationships are shown below. Base-pair states of site 17 are indicated using different colors in the matrix and are mapped and optimized on the tree to exemplify an unambiguous CBC process. (**c**) Representative sequence-structures showing different base-pair states of site 17. Numbers following a species name indicate GenBank accession numbers.

CBCs are commonly observed in closed related species. For example, an AU base pair in the ITS2 secondary structure of one species and a GC base pair in the homologous position of the other species (Figure 2c). A better understanding of this base-pair-change pathway along the evolutionary process is essential to give an accurate ITS2 evolutionary model. Since mutation is rare in natural organisms, it is very likely that two substitution events occur separately rather than simultaneously, through which an intermediate base pair must exist. There are three possible cases for a given base of a base pair changing to another form. For example, an AU base pair could change to AC, AG or AA intermediate. However, given that transition is easier than transversion, AU would more likely change to AC than AG or AA. In addition, these intermediates are assumed under negative selection due to the decrease of structure stability and could be eventually eliminated from the population. However, the AC intermediate is most likely fixed because it is more stable than AG and AA and less selectively disadvantaged [42]. Similarly, A on the other side is more likely to change to G rather than U or C. Taken together, an AU change to GC is generally considered through two-step transition with AC as the intermediate. Taking into account the other purine–pyrimidine GU, there are eight possible pathways in all for CBC substitution with ITS2 secondary structure [42].

Notably, different intermediates (GU and AC) in the same RNA region may exist under different selection strengths. If the selective disadvantage is slight, the intermediates can exist with high frequency in the population. In contrast, if the selective disadvantage is strong, the intermediates are always transient with low frequency before the second substitution occurs. In this case, the two substitution events of CBC occur so rapidly that they appear to occur simultaneously. We found that the average frequency of AC is only one-seventh of GU, but the average mutability of AC is nearly twice as much as that of GU. Therefore, we caution that if AC is neglected in compensatory mutation, these CBCs could easily be misinterpreted as simultaneous double substitutions [42].

## 8. CBCs Character Weighting

Phylogenetic analyses of ITS2 are exclusively based on its nucleotide sequences and DNA evolutionary models, which assume that sites in a sequence evolve independently of each other. However, this is not the case for RNAs, in which the core secondary structures are maintained by CBC under functional constraints. Some authors are concerned that failing to account for CBC substitutions is equal to counting the same variation twice and can lead to misleading phylogenetic inferences with strong support. To eliminate CBC effects, a simple one-half weighting of paired positions in animal 5S rDNA has been suggested [48]. In contrast, Dixon & Hillis recommend that weighting of stem positions should be no more than 20% in animal 28S rDNA [49]. The discrepancy of this relative weighting of stems and loops may be due to the difference of frequencies and patterns of CBCs in different rDNA regions. For example, 5S rDNAs are estimated with the highest rate of CBC and thus comprise the largest number of non-independent characters. In this case, variation in stems should be given lower weights than loops to reduce the effect of CBCs. Notably, these results indicate that weighting variations of stems and loops for phylogenetic analyses could be specific to gene regions or datasets, making it difficult to set a priori weights with general significance.

How much of the co-variable sites are present in the ITS2 region and to what extent do they affect ITS2 phylogeny? We recently examined this issue within a more recent lineage of the plant *Corydalis*. We first partitioned the ITS2 sequence into paired and unpaired regions based on the known structure, and assigned them a RNA and DNA model, respectively. We found that the G+C content in the paired regions is 1.5-fold higher than that of the unpaired regions. In contrast, the adenine in the unpaired regions is 4.4-fold that of paired regions. These nucleotide composition differences are consistent with the expectation of functional constraint where selection tends to maintain the thermodynamically stable secondary structure, and the adenine positions in single strand are associated with long-range interactions of tertiary structure [50]. In addition, we found that the variable rate was lower but the Ts/Tv ratio was higher in paired versus unpaired regions, indicating that their substitution pattern was also distinct. Given that 60% of the ITS2 nucleotides are involved in stem pairing together with these differences in nucleotide frequencies and substitution bias, structural-partition and paired-site RNA models may be more appropriate for the ITS2 region than standard DNA models. Of the 137 variable sites, we observed 22 (16%) sites involving compensatory mutation, but only two CBCs (3% sites) were detected, by which non-independent variation is unambiguous. Consequently, the empirical effect of the site-independence violation is slight in this study as it is based on closely related species, in contrast to the more severe effects that have been reported in more ancient lineages [48,49]. We thus highlight that although site variation in stem could violate the site-independence assumption, double compensatory changes (CBC) rather than all compensatory changes (including hemi-CBC) should be half down weighted in phylogenetic analyses. This result also emphasizes the importance of future study to explore at what taxonomic level or degree of sequence divergence should the CBC effect be considered in phylogenetic analyses, since more and more hemi-CBCs could change to CBCs as the sequence divergence increases.

## 9. RNA Substitution Models

Since the evolutionary constraints are imposed on the structure rather than on the nucleotides themselves, a number of RNA substitution models have been suggested to consider the paired site as a functional unit rather than treat both stem sides separately [46,51]. In principle, there are 16 possible combinations that can be formed by four bases, and thus a 16 × 16 substitution rate matrix is needed to describe the evolutionary process of the paired regions from one state to another. However, such an over-parameterized model is too computationally time-consuming to be used in practice. A possible solution to this problem is to reduce the base-pair state to the six base pairs (GC, CG, AU, UA, GU and UG) that occur frequently and to disregard the remaining 10, so called mismatch (MM) pairs that occur rarely. The 6-state models completely disregard MMs while the 7-state models lump them together into a single state MM [46]. Another simplification of the substitution rate matrix is the reduction of base-pair frequency parameters by imposing base-pair reversal symmetry. For example, the frequency of GU is expected equally as UG. Similarly, base-pair rates between different base pairs can also be generalized by single transition substitution between WC pair and the intermediate GU/UG, double transition substitution between AU and GC, double transversion substitution between purine-pyrimidine and pyrimidine-purine, and substitution to and from a MM state (if any), or by further setting all double-substitution rates to zero [42]. Taking all these constraints together, a total of 18 RNA substitution models have been summarized by Savill et al. [46]. Notably, by assuming that the substitution process within a given lineage is constant, equilibrium frequency and rate parameters can be defined, including substitutions involving MM. Thus, the transient base pairs and their frequencies in RNA structure can be detected which has always been difficult with the conventional biochemical-detection methods [42].

## 10. Species Delimitation Based on ITS2-CBC

In addition to phylogenetic utility of substitution models of CBC, another potential use of ITS2 is species delimitation based directly on CBC numbers. In their study of ITS2 among species of green algae *Volvocales*, Coleman and colleagues defined “Z clade” as a group of individuals producing compatible gametes and, correspondingly, they defined “CBC clade” as a group of individuals sharing no CBC within them but that have at least one CBC between other CBC clades in their ITS2 conserved regions [52]. Intriguingly, different CBC clades seem always nested within different Z clades, indicating that CBC correlates with the inability to sexually cross, which is also used as a biological species definition [53]. Müller et al. tested this hypothesis on a broad range of plants and fungi using the large dataset of ITS2 database [54]. Their results showed that two organisms that differed by a CBC in ITS2 have a 93% probability of being a distinct species. In contrast, two organisms that lack CBC have a 76% probability of being the same species. Given that the fast-evolving ITS2 could vary within the intra-genome, intragenomic CBC could blur species boundary. Wolf et al. showed that intragenomic CBC is very rare (0.01%) in ITS2 and has a minimal effect on CBC species concept [55]. Based on the above evidence, wherein CBCs occur between rather than within species, Wolf et al. proposed a generalized “CBC species concept” [55]. Further development of Coleman’s hypothesis showed that CBC should expand to all four helices and even hemi-CBC rather than being restricted to helix II and III as Coleman originally suggested [43,56,57]. In practice, rapid identification of the ITS2 CBC among the potential parental species and assessing their fertility before their actual cross experiment could reduce the incompatible mating pairs and save time. Thus the CBC species concept could facilitate plant breeding.

Although CBC exhibits great potential use for species distinction, some authors caution that CBC should not be expected to distinguish all species from each other. For example, only 25 out of 98 morphological species of foliar endophytic fungi were differentiated by CBC characters [58]. Likewise, only two CBCs are present among ten closely related species of *Corydalis* [43]. These results are consistent with some previous findings that CBC occurrence primarily corresponds to higher supra-specific lineages rather than species level distinction [57,59]. At present, there is no evidence of functional correlation between ITS2 sequence and gamete fusion and ITS2-CBC by itself is not related to speciation. The molecular dating of *Corydalis* showed that ITS2-CBC substitutions were separated by ~10.4–15.5 million years, within which several species have occurred [43]. This result indicates that CBC can provide a scale for measuring whether sufficient evolutionary time has elapsed for a speciation event in certain lineages.

## 11. Determining CBCs and Their Substitution Processes

If CBC is the proxy of evolutionary time for a speciation event, some caution should be taken when using it. First, only two or a more speciation events could be accompanied by CBC change from AU to GC, and then to AU again. Therefore, substitution reversals likely occur in ancient lineages. On the other hand, in most ancient-lineage phylogeny, wherein a single sequence often represents an entire species, low-frequency base-pair states generally cannot be observed [60].

To avoid misused CBCs, we suggest evolutionary changes occurring in ITS2 secondary structure should be mapped onto the phylogeny using sufficient inter- and intraspecific sampling. As developed in our previous studies [42], ITS2 sequences are first folded and aligned synchronously into a sequence-structure alignment by LocARNA [61]. Alternatively, each ITS2 secondary structure can be predicted individually by the homology modeling method (Figure 2c) [19], and then align the sequence-structure matrix by 4SALE [32]. Both methods could equally generate a consensus secondary structure of all investigated ITS2 sequences (Figure 2a). In such sequence-structure alignment, each base pair is treated as a unit and transformed into a 28-character coding matrix based on the consensus secondary structure (Figure 2b) [40]. Meanwhile, a phylogenetic tree can be constructed from the sequence-structure alignment by using a DNA/RNA mixed model (DNA model for the unpaired regions and RNA model for the paired regions) implemented in the PHASE 3.0 package [51]. Given a set of base-pair characters and a phylogenetic tree, both from the sequence-structure alignment, the base-pair states involving CBC can be optimized on the inferred tree by using Fitch optimization (Figure 2b) [62]. Unlike the CBC-counting program, CBCAnalyzer [63], this method shows not only the total number of CBCs, but the base-pair states in each site and their evolutionary processes. Therefore, this visualized CBC-determining method allows more precise quantification of diverse CBC types and their substitution processes.

## 12. Concluding Remarks

ITS2 is an ideal material for the integrative study of function, structure and evolution. As a widely used phylogenetic marker, ITS2 sequence has experienced explosive growth in the last twenty years, making it the gene region with the largest sequence number in GenBank. Notably, ITS2 has its own sequence-structure database, The ITS2 Database. The availability of huge amounts of ITS2 data across a broad eukaryotic diversity will facilitate this integrative study. In addition, as part of the study of ribosome biogenesis, the structure and function of ITS2 has been intensively explored for more than thirty years using multiple methods of chemical and structural probing, electron microscopy, site-directed mutagenesis and free-energy algorithms. At present, the structure and function of ITS2 has become increasingly clear. This information contributes greatly to the study of ITS2 evolutionary studies. For example, functional studies demonstrate that interaction between the 3′ end of 5.8S and the 5′ end of 28S form a conserved structure of an ITS2-proximal stem in ribosome biogenesis. This finding has been successfully applied to delimit ITS2 boundary in ITS2-related evolutionary studies. Furthermore, the characteristics of ITS2 conservative secondary structure have been used in the alignment of divergent ITS2 sequences, by which phylogenetic analyses of ITS2 can be at higher taxonomic level. In turn, evolutionary analyses of nucleotide sequences can facilitate structure prediction. For example, the site covariation (compensatory base change) in combination with phylogenetic comparison is increasingly used as a more effective method for structure prediction. Incredibly, more than 97% base pairs predicted in 16S and 23S ribosomal RNA using a comparative analysis method were found in the crystal structures, indicating that the rationale and method we review herein could expand to other ribosomal regions.

## Abbreviations

AFs	assembly factors
cryo-EM	cryo–electron microscopy
CBC	compensatory base change
ETS	external spacer
ITS	internal transcribed spacers
LSU	large subunit
MEF	minimum free energy
RPs	ribosomal proteins
rRNA	ribosomal RNA
SSU	small subunit
